# Nootropics as Cognitive Enhancers: Types, Dosage and Side Effects of Smart Drugs

**DOI:** 10.3390/nu14163367

**Published:** 2022-08-17

**Authors:** Matěj Malík, Pavel Tlustoš

**Affiliations:** Department of Agroenvironmental Chemistry and Plant Nutrition, Czech University of Life Sciences Prague, Kamýcká 129, 165 00 Prague, Czech Republic

**Keywords:** learning, smart drugs, brain injury, piracetam, *Panax ginseng*, nootropics, memory, *Paullinia cupana*, antioxidant activity, ayurvedic

## Abstract

Nootropics, also known as “smart drugs” are a diverse group of medicinal substances whose action improves human thinking, learning, and memory, especially in cases where these functions are impaired. This review provides an up-to-date overview of the potential effectiveness and importance of nootropics. Based on their nature and their effects, this heterogeneous group of drugs has been divided into four subgroups: classical nootropic compounds, substances increasing brain metabolism, cholinergic, and plants and their extracts with nootropic effects. Each subgroup of nootropics contains several main representatives, and for each one, its uses, indications, experimental treatments, dosage, and possible side effects and contraindications are discussed. For the nootropic plant extracts, there is also a brief description of each plant representative, its occurrence, history, and chemical composition of the medicinal part. Lastly, specific recommendations regarding the use of nootropics by both ill and healthy individuals are summarized.

## 1. Introduction

At one time or another, everyone has dreamed of becoming more intelligent, learning more things in less time, thinking and reacting faster, and having a better memory. There are compounds currently available on the market that promise various combinations of the benefits mentioned above. This group of substances is known as the nootropics [1]. Although these substances are more effective in cases where cognitive functions are obviously impaired, they are of interest to healthy individuals because of their ability to increase intelligence and improve memory [2]. The vast majority of these substances are of natural origin [3], not subject to prescription, and can usually be easily obtained in the form of food supplements or herbal extracts. Their availability in synthetic form is somewhat limited and some preparations do require a valid prescription to obtain them. Nootropics tend to be well tolerated in patients with cognitive impairments; the incidence of side effects is low, and those that do occur are usually mild [4,5,6]. Most nootropics do not have an immediate effect after a single dose, and therefore long-term use is necessary to achieve the desired results [7]. However, their long-term effects on healthy individuals are still unknown [8].

This literature review provides an overview of the potential importance of nootropics, their types, use, dosage, and side effects. Original research articles, meta-analyses, and systematic reviews were included in our investigation and relevant animal studies were also considered. We did not limit our review to specific results, but focused on providing an up-to-date overview of readily available substances, primarily over-the-counter, either as food supplements or medications, that are also used by healthy people such as students. We tried to include all the currently popular “smart drugs.” Illegal drugs and drugs with a primarily non-nootropic function, such as stimulants, vitamins, etc., were not included. There have been few studies on healthy young individuals, so we tried to describe the effects of these substances also on individuals whose cognitive functions were impaired. Lastly, we summarized their potential effectiveness with recommendations for use.

## 2. What Are Nootropics?

Nootropics, also known as “smart drugs” in English language journals [2], are a heterogeneous group of compounds [9]. The term “nootropic” was first used by Cornelius E. Giurgea in 1972/1973 [10,11] to describe substances that primarily activate cognitive functions, such as memory and learning, especially in situations where these functions are impaired [1]. In a sense, they interfere with the metabolism of neuronal cells of the central nervous system (CNS) [12,13,14]. The name consists of two Greek words: *nöos*, which means thinking, and *tropein*, which means to guide [10,11]. There is no uniform approach to categorizing these compounds. Some authors distinguish between classical nootropics and substances that enhance brain metabolism, while others combine these two groups, or use the term cognitive effect rather than nootropic [15]. 

### 2.1. Mechanisms of Action

Nootropics do not act directly by releasing neurotransmitters or as receptor ligands [16], but improve the brain’s supply of glucose and oxygen, have antihypoxic effects, and protect brain tissue from neurotoxicity [9,17]. They also positively affect neuronal protein and nucleic acid synthesis and stimulate phospholipid metabolism in neurohormonal membranes [18,19]. Some nootropics have been found to affect the elimination of oxygen free radicals, possess an anti-aggregation effect, and improve erythrocyte plasticity. This improves the rheological properties of the blood and improves blood flow to the brain [3,20,21]. These substances are metabolically active, but most nootropics show no immediate effects after a single dose, requiring an extended period of use to produce results. They need to be able to penetrate the blood–brain barrier to improve brain metabolism and long-term use is necessary to achieve stable changes [7].

### 2.2. Indications

Nootropics are used in acute or subacute conditions for treating memory, consciousness, and learning disorders [22]. They are recommended for incipient brain damage, which manifests with memory loss, mental retardation, and qualitative changes in consciousness. This condition is referred to as acute psychoorganic syndrome (POS). It is usually reversible, but it can progress to dementia in some cases. Acute POS can be caused by brain trauma, infection, stroke, or intoxication (alcohol, drugs with central anticholinergic effect, or carbon monoxide). Delirium tremens also belong to the POS group [23]. 

Other indications may include chronic disorders of cognitive functions such as mental retardation or memory impairment [22]. Nootropics are given relatively often in these cases, but their benefit, especially in more severe dementia, is questionable. They seem to be more effective in patients with mild cognitive disorders or the so-called benign senescent forgetting when there is only a slowing down of brain function without the development of dementia [24,25]. Nootropics are sometimes used for attention and memory disorders due to fatigue and exhaustion [26,27]. They are also used by children with minimal brain dysfunction syndrome [28,29] and patients with encephalopathy [30], and their effect on myalgic encephalomyelitis (chronic fatigue syndrome) has also been tested [31]. As cognitive enhancers, nootropics are administered to patients who have Alzheimer’s disease [3,32], schizophrenia [33], hyperkinetic disorder [34,35], or senile dementia [15,24,25].

### 2.3. Nootropic Treatment

Nootropics are usually very well tolerated. Their efficacy depends on the size of the dose, and in practice, administering too low a dose is a common mistake. Treatment should be continued for at least 2–3 weeks after the disturbance of consciousness has disappeared [10]. A clinical scale has been developed to assess the depth and duration of impaired consciousness. Three aspects of behavior, such as motor response, verbal performance, and eye-opening, are measured independently. These are recorded and consistently evaluated according to the chart [36]. Side effects of nootropics are uncommon and are rarely serious. In addition to individual intolerance, an increase in activity in the undesired direction, a sleep disorder, or an increase in libido may occasionally occur [1,4,5,6]. Nootropics are contraindicated in hypersensitivity, pregnancy, and lactation [6].

### 2.4. Use by Students

Nootropics, thanks to their alleged ability to increase intelligence and improve memory and cognitive functions, attract the attention of university students in particular. They are known among them as ‘smart drugs’ [8,37]. Because most nootropics are of natural origin, students can obtain them as food supplements or as drugs that do not require a prescription [3], and, like many other substances and drugs, nootropics are increasingly available on the Internet. However, the use of nootropics by healthy individuals is of great concern due to the lack of clinical evidence regarding their efficacy, safety, and social consequences, especially in long-term use [2,8].

### 2.5. Advantages and Disadvantages of Natural vs. Synthetic Nootropics

The indisputable advantage of natural origin drugs from several plant organs (flower, leaf, root, etc.), is that they can have a greater variety of potentially beneficial pharmaceutical effects. This is due to the diverse composition of substances in a herbal drug that can have synergistic or additive effects [38]. Natural nootropics also usually have lower toxicity, which reduces the possibility of harm from an overdose. However, some compounds can reduce the pharmaceutical activity of other compounds [39]. Higher doses of such a herbal drug are needed to achieve the desired effect, which is why plant extracts are often used. There is also a problem in the case of storage or possible falsification and verification of the authenticity [40,41]. The advantages of synthetic compounds are their pharmaceutical purity, specificity of action, and a possible increase in their effect by modification of the chemical structure [42]. They usually are active at lower dosages, but this entails a greater risk of overdose [43].

## 3. Classical Nootropic Compounds

### 3.1. Deanol (DMAE)

**Figure 1:**
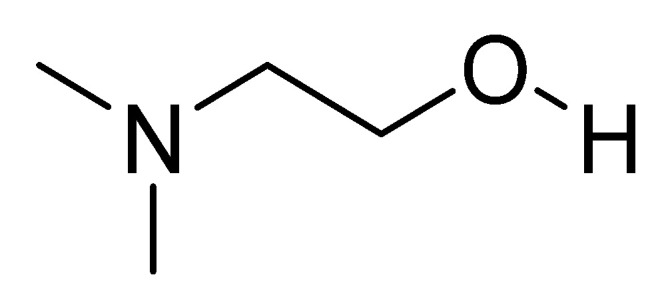
Chemical structure of deanol.

The chemical name is 2-(dimethylamino)ethan-1-ol and the chemical structure is shown in Figure 1 [44]. The compound is physiologically present in the human brain. Deanol is commonly marketed as a natural dietary supplement. Many nutritional supplements contain DMAE in the form of the salt of tartaric acid (bitartrate salt). Small amounts can also be obtained from eating fish, especially salmon and shellfish. Deanol is a choline precursor that allows the brain to optimize the production of acetylcholine, the primary neurotransmitter involved in learning and memory [45]. 

Dimethylaminoethanol pyroglutamate increased choline and acetylcholine extracellular levels in the brain’s prefrontal cortex in vivo in rat experiments. It further improved spatial memory and reduced scopolamine-induced memory deficits [46]. Dimethylaminoethanol cyclohexyl carboxylate fumarate significantly enhanced working memory performance in rats in the radial arm maze [47]. 

According to an electroencephalogram (EEG) analysis, supplements combining vitamins and minerals with compounds containing DMAE in humans for three months showed increased alertness, attention, and overall mood improvement [48]. DMAE also improved sleep quality and was able to induce lucid dreams [49]. Its administration has been tested in child hyperkinetic syndrome [50] and minimal brain dysfunction syndrome [51]. 

The daily dosage should be 500–2000 mg in the form of DMAE bitartrate [52,53]. It is contraindicated during pregnancy, lactation, and in patients with schizophrenia [50].

### 3.2. Meclofenoxate

**Figure 2:**
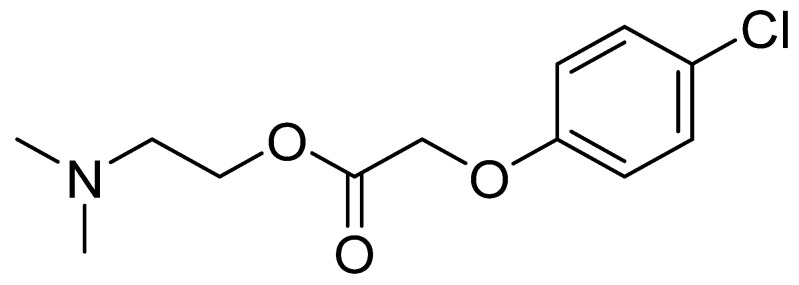
Chemical structure of meclofenoxate.

The meclofenoxate molecule consists of two parts (Figure 2). The first part is a synthetic auxin, a 4-chlorophenoxyacetic acid similar to the natural auxin indoleacetic acid found in plant cells and which acts to exchange carbohydrates. The second part of the molecule consists of the already mentioned 2-(dimethylamino)ethan-1-ol or deanol [44]. 

Meclofenoxate is well absorbed when administered parenterally. It dramatically increased CNS choline levels in vivo (in rats). In the hippocampus, this increase in choline was also accompanied by an increased level of acetylcholine. Thus, its effects on choline and acetylcholine levels in the brain are similar to those of deanol but appear to be about twice as effective [54]. Oral administration of meclofenoxate to rats (100 mg/kg, daily for 37 days) significantly improved memory impairment, and reduced neuronal damage, proinflammatory mediator levels, and oxidative stress to normal levels. The ability to alleviate memory deficits and neuronal damage may benefit cerebrovascular dementia [55]. The RNA-Seq study of brain tissues of Nothobranchius guentheri, which received meclofenoxate for almost a lifetime, concluded that while meclofenoxate compensated for age-dependent downregulation of neuronal activity genes, its effect on the aging brain transcriptome still could not be considered unequivocally positive [56].

In a double-blind study, meclofenoxate also increased mental alertness and consolidation of new information into long-term memory in elderly people [57]. It may be a useful therapeutic tool for potentiating depressed cholinergic neurons and treating neuroleptic-induced dyskinesias [58]. Meclofenoxate improves the status of qualitatively altered consciousness, has an antihypoxic effect, and is used to mitigate the overall slowdown in speech, thinking, and mental activity caused by CNS intoxication and injury. It has also been tested for treating Alzheimer’s disease and vascular dementia [59]. 

The daily dose should be 500–2000 mg [58]. Meclofenoxate is deemed to be safe and tolerable. Possible side effects are often caused by overdose, including dizziness, restlessness, nausea, and headache [58,60].

### 3.3. Nicergoline

**Figure 3:**
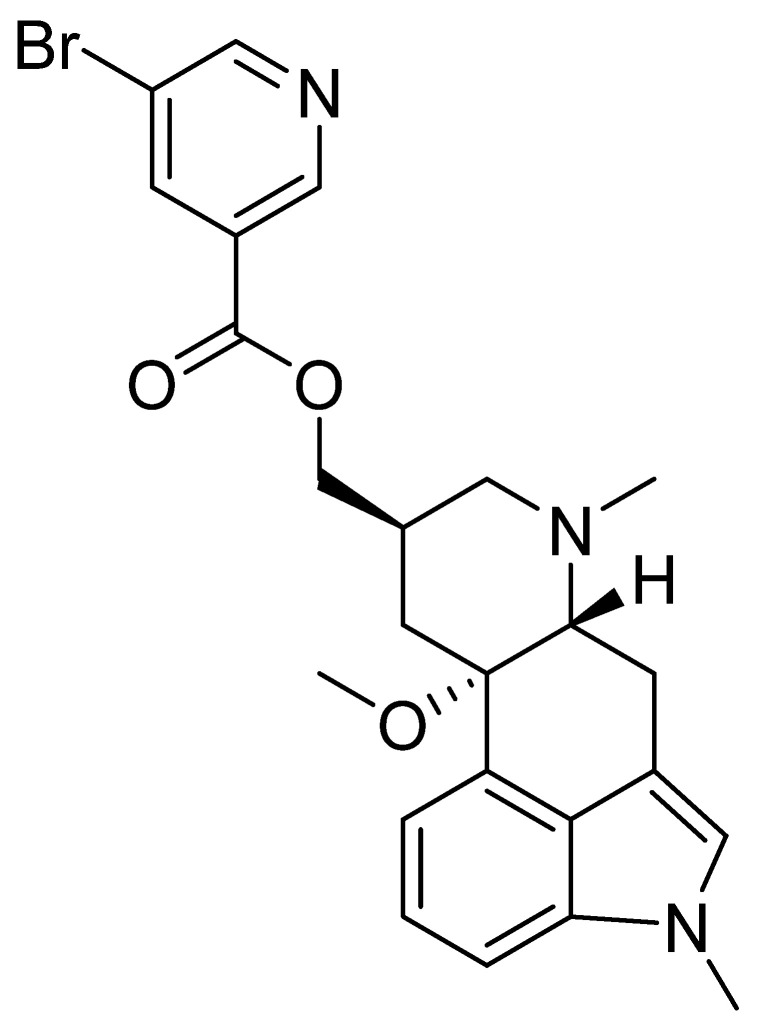
Chemical structure of nicergoline.

Nicergoline is an ergot alkaloid, also known as nicergoline (Figure 3) that has been clinically used since 1970. Nicergoline was initially developed as a vasodilator prescribed for cerebrovascular disorders. It is currently used clinically to treat syndromes of vascular or degenerative origin characterized by cognitive impairment with decreased intellect, and affective, behavioral, and somatic disorders. Specifically, it is used for memory loss, reduced alertness, concentration ability, mood swings, dizziness, fatigue, and vestibular and cochlear disorders [61,62]. 

Nicergoline has a wide range of effects. It protected cultured neurons from β-amyloid toxicity in vitro [63]. Nicergoline has been shown to be an effective drug for preventing neuronal vulnerability due to experimentally induced nerve growth factor deprivation and improved the function of cholinergic and catecholaminergic neurotransmitters in rats in vivo [64]. It acted as an antagonist of α_1_-adrenoceptors [65], increased arterial blood circulation [66], inhibited platelet aggregation, supported metabolic activity (resulting in increased oxygen and glucose utilization), and had neurotrophic and antioxidant properties in rats in vivo [67]. Nicergoline also showed an improving effect on cognitive function in mouse models of Alzheimer’s disease [68].

Nicergoline induced vasodilation and increased cerebral blood flow [69]. Its efficacy has also been demonstrated in patients with vascular dementia [70]. Nicergoline showed a comprehensive positive effect on different levels of cerebral, systemic, and cardiac hemodynamics in ischemic stroke patients [71]. According to an electroencephalogram/event-related potential (EEG/ERP) mapping study, in patients with multi-infarct dementia and Alzheimer’s disease, nicergoline improved alertness and information processing at the neurophysiological level, which led to clinical improvement at the behavioral level in both degenerative and vascular dementia [72]. 

The daily dose should be 30–60 mg [62]. Side effects are rare and usually include nausea, dizziness, diarrhea, fainting, and headache [73,74]. Due to the lack of experience with nicergoline in pregnant women, it is not recommended during pregnancy and lactation [74].

### 3.4. Piracetam

**Figure 4:**
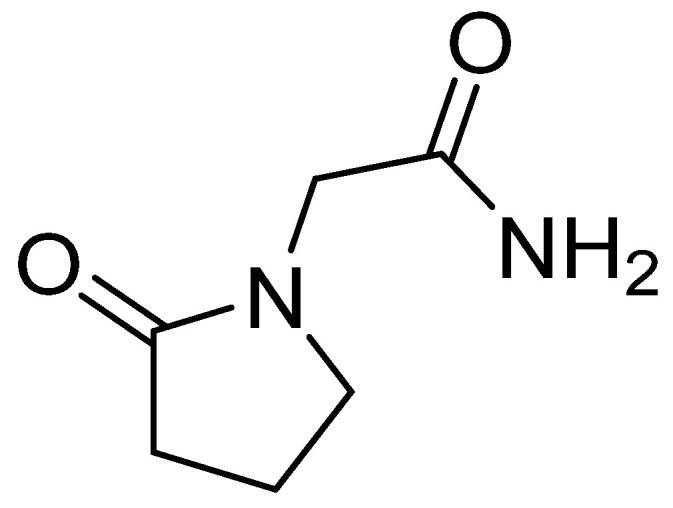
Chemical structure of piracetam.

The chemical name of piracetam is 2-(2-oxopyrrolidin-1-yl)acetamide (Figure 4). It is a cyclic derivative of gamma-aminobutyric acid (GABA) and acetamide. Piracetam is thought to act on brain neurotransmission through modulation of ion channels (Ca^2+^ and K^+^), leading to nonspecifically increased neuronal excitability [75]. 

It enhanced the function of the neurotransmitter acetylcholine via muscarinic receptors [76], and affected N-methyl-D-aspartate receptors in rat models in vivo, increasing cell membrane permeability [77]. Piracetam has also been found to increase oxygen consumption in the brain and, in connection with adenosine triphosphate metabolism, it increased adenylate kinase activity in vivo in the rat brain [78]. It appears to increase the synthesis of cytochrome b_5_ [79], which is involved in the mechanism of electron transport in mitochondria, where it also increases permeability. It alleviated the intensity of hypoxia-induced nerve cell damage, improved interhemispheric transmission, and increased glucose metabolism in the rat brain [80]. Piracetam has been tested for stroke, unconsciousness, treatment of withdrawal symptoms from alcoholism, and prevention of alcohol-induced hypoxia [81,82]. It also improved brain function in rat models affected by xenobiotics [83].

In addition to the metabolic effect on brain tissue, piracetam enhances erythrocyte plasticity and consequent brain perfusion [84]. It was used clinically for the prevention and treatment of post-traumatic cognitive and mental dysfunction, and to improve learning and memory functions in developmental dyslexia in children patients [85]. Piracetam has also been tested for treating Alzheimer’s disease [86] and combined with lecithin [87], but unfortunately without significant benefit in patients. The structural analogues of piracetam are oxiracetam, pramiracetam, etiracetam, nefiracetam, and aniracetam. These compounds act similarly to piracetam, but with varying efficacy [42,75]. According to the assessment of the effectiveness of nefiracetam on higher brain functions in terms of time and space using electric field distribution of the scalp map and low-resolution electromagnetic tomography for evoked potentials and spontaneous EEG with eyes closed, the Gottfries-Brane-Steen scale showed significant improvement. However, the Mini-Mental State Examination, the Hasegawa Dementia Scale, and the Kohs block test showed no improvement. These results suggest that nefiracetam has some benefits in patients with vascular dementia [88]. 

The effective dose of piracetam as an infusion for acute treatment is 4–8 g per day. The maintenance dose is usually about 2–4 g/day, adjusted based on kidney function. The tolerance of piracetam is excellent with only rare side effects, including insomnia, irritability, increased libido, and sexual function [75,83]. There is insufficient clinical experience with piracetam in pregnancy. Animal studies have not shown teratogenic or other embryotoxic effects, but piracetam should still be used during pregnancy only after carefully weighing the expected benefits against the potential risks. It should not be used during lactation [89].

### 3.5. Pyritinol

**Figure 5:**
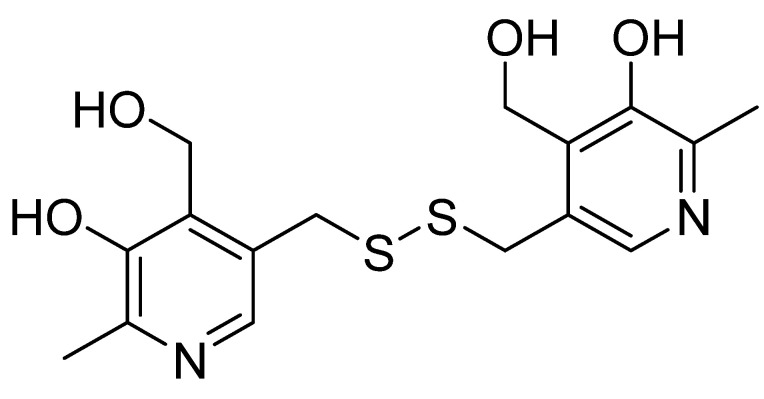
Chemical structure of pyritinol.

Additionally known as pyridoxine disulfide or pyrithioxin (Figure 5), pyritinol was synthesized in 1961 by combining two molecules of vitamin B_6_ (pyridoxine) via a disulfide bridge. Like pyridoxine, it has different effects on different organ systems; however, the CNS is the primary system in which pyritinol exhibits observable pharmacological effects. Pyritinol crosses the blood–brain barrier and accumulates in gray matter, especially in the hippocampus, cerebral nuclei, cerebellum, and cortex [90]. 

Animal studies have demonstrated effects on various neurotransmitters [91]. In vivo assays in rats revealed increased choline acetyltransferase activity, leading to choline accumulation in cholinergic neurons [92]. Pyritinol plays a supporting role in the recovery of age-related brain deficits. For example, in elderly rats, pyritinol metabolites increased cortical acetylcholine concentration and release, and nucleic acid metabolism in the brain [93]. Acute or prolonged oral administration of pyritinol reduced formaldehyde-induced nociceptive behavior and tactile allodynia in old diabetic rats. Pyritinol was also able to scavenge oxygen free radicals, thus acting as an antioxidant and improving cerebral circulation [94]. It also restored the decreased concentration of the primary excitatory neurotransmitter N-methyl-D-aspartate when administered to older mice [95]. Results of the experiment on rats showed that pyritinol may be helpful in learning and memory disorders caused by malnutrition and deprivation [96]. 

A study in healthy human males treated with pyritinol showed performance improvements in response time tests but not in memory tests [97]. Intramuscular injections of nandrolone decanoate and pyritinol have dramatically affected motor development and learning ability in children with cerebral palsy, without side effects. The combined effects of vinpocetine and pyritinol also improved blood and plasma viscosity in human patients with cerebrovascular disorders [98]. Due to the status of pyritinol as a dietary supplement in some countries, it can be safely used as an adjunct to any standard treatment of CNS diseases, such as developmental dysphasia and other cognitive disorders, for which current therapeutic options are limited [90]. 

No accumulation of the substance was observed, even after repeated oral administration, and toxic concentrations were not reached even in patients with impaired renal function. In practice, underdosing is common. The minimum recommended daily dose is 300 mg, divided into three sub-doses, but the amount taken should be 600 mg or more [90,97]. The common side effects are non-specific rashes, headache, inflammation of the oral mucosa, acute pancreatitis, diarrhea, nausea, and loss of appetite [99,100,101]. Pyritinol crosses the placenta, but systemic testing in mice and rats did not show teratogenic or embryotoxic effects. Only minimal amounts of pyritinol are excreted in human milk but a careful evaluation should still be made before dosing during pregnancy and lactation [90,101].

## 4. Substances Increasing Brain Metabolism

This group of substances exhibits simultaneous nootropic, hemorheological, and vasodilatory effects. Examples include vinpocetine, naftidrofuryl, and dihydroergotoxine, which is a mixture of dihydrogenated ergot alkaloids [102,103,104,105,106].

### 4.1. Vinpocetine

**Figure 6:**
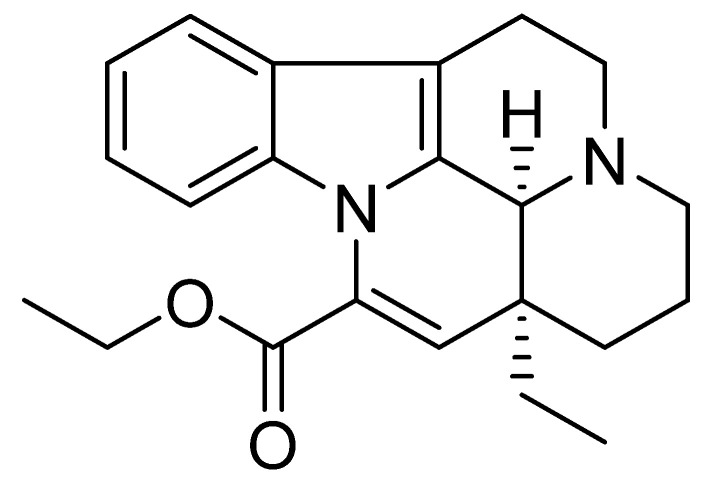
Chemical structure of vinpocetine.

Vinpocetine (Figure 6) is a semisynthetic derivative of the vincamine alkaloid that occurs in the lesser periwinkle, Vinca minor [107]. 

Experiments ex vivo have shown that vinpocetine acts as a selective inhibitor of Ca^2+^/calmodulin-dependent cyclic nucleotide phosphodiesterase type I [21,108], a potent blocker of voltage-gated sodium channels [109,110], inhibits platelet aggregation, reduces blood viscosity, vasodilates cerebral arteries, and increases cerebral blood flow [111]. Ex vivo, vinpocetine increased glucose and oxygen consumption through brain tissue and improved brain cell tolerance to hypoxia [112]. 

In vitro, vinpocetine interacted with glutamate receptors [113], shifted glucose metabolism to more energy-efficient aerobic processes, and increased adenosine triphosphate (ATP) levels in the brain [114]. Thus, vinpocetine offers significant and direct neuroprotection in vitro and in vivo [104]. This vasoactive alkaloid has been marketed for several years as an adjunct to vasodilators and nootropics to improve memory [115]. It is also considered an active substance in treating stroke and other diseases, including circulatory disorders in the brain [116,117]. 

It is recommended that users take only 2–5 mg for the first time to ensure that they do not have a hypersensitive reaction to it. They can then increase the dose to 10–30 mg daily [118,119], a dose that can cause some side effects, although very rarely, including nausea, dry mouth, dizziness, headache, and heartburn [120]. The use of vinpocetine is contraindicated during lactation and pregnancy [121].

### 4.2. Naftidrofuryl

**Figure 7:**
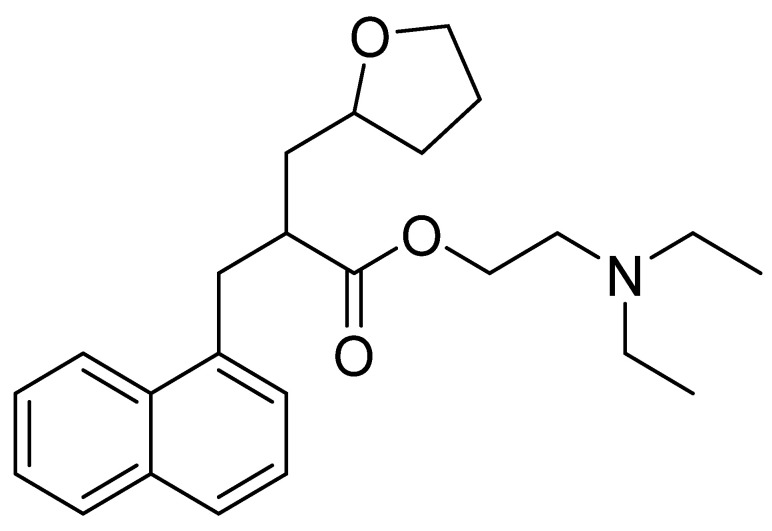
Chemical structure of naftidrofuryl.

Chemically, naftidrofuryl is a 2-(1-naphthalenylmethyl)-3-(2-oxolanyl)propanoic acid ester of 2-(diethylamino)ethanol (Figure 7). Naftidrofuryl is a vasodilator with beneficial rheological effects on the blood and has long been used to treat intermittent claudication to improve walking and provide symptomatic relief [103]. 

Naftidrofuryl in vitro has shown a regulatory impact on deoxyglucose uptake [122] and glucose utilization [123], and it inhibited the hypoxia-induced decrease in ATP levels in fibroblasts and endothelial cells in vitro [124]. It inhibited serotonin- and epinephrine-induced platelet aggregation in vitro and ex vivo [125,126]. 

In mouse brains, it showed an antagonistic effect on 5-HT_2_ receptors of vascular smooth muscle cells and platelets and inhibited serotonin-induced contractions in blood vessels [127]. 5-HT_2_ receptors are a subfamily of 5-HT receptors that bind the endogenous neurotransmitter serotonin (also called 5-hydroxytryptamine, 5-HT). Serotonin is important in vasoconstriction and platelet aggregation, leading to atherosclerosis [128]. Naftidrofuryl was then shown to have antiatherosclerotic effects in various animal models [129,130]. In rats, it also increased the storage of spatial information and showed nootropic effects [131].

In a double-blind study in human volunteers, naftidrofuryl increased erythrocyte deformability and flow [132]. The induced reduction in the lactate/pyruvate ratio in healthy human volunteers during exercise suggests that naftidrofuryl increases the efficiency of aerobic metabolism in oxygen-deprived tissues [133]. It also has a positive effect on the energy metabolism of the neuron. Naftidrofuryl is used in the treatment of cardiovascular diseases [134], senile dementia [135], and Alzheimer’s disease [136]. 

To treat patients with mild to moderate occlusive peripheral arterial disease, it is recommended that naftidrofuryl be administered orally at a dose of 300 to 600 mg/day in three divided sub-doses, swallowed whole. Naftidrofuryl metabolism may be reduced in elderly patients. Therefore, the dose may need to be lowered for these patients [102,103]. Naftidrofuryl is well tolerated, and side effects occur only rarely. These are usually gastrointestinal problems, but there was a single known case of liver damage [137]. 

### 4.3. Dihydroergotoxine

**Figure 8:**
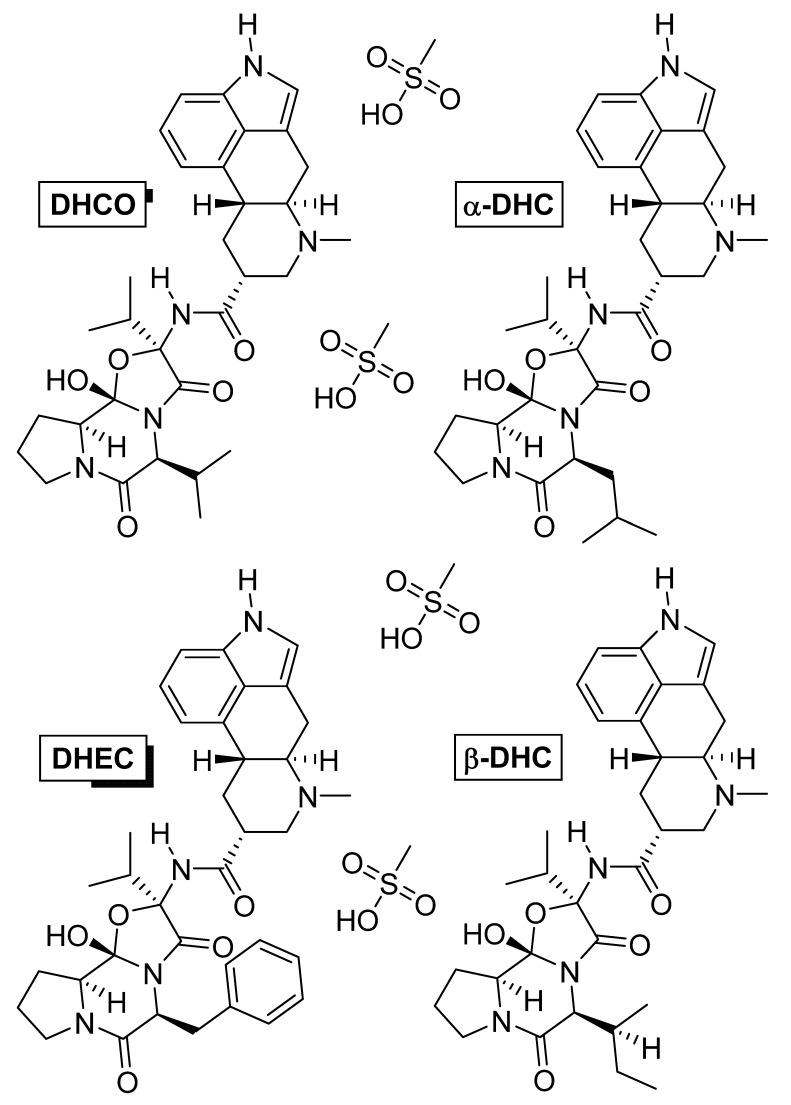
Chemical structures of the methanesulfonate salts comprising dihydroergotoxine. Abbreviations: DHCO, dihydroergocornine; DHEC, dihydroergocristine; α-DHC, alpha-dihydroergocryptine; β-DHC, beta-dihydroergocryptine.

Dihydroergotoxine (Figure 8) is also known as hydergine or ergoloid mesylate, and is a mixture of the methanesulfonate salts of dihydrogenated ergot alkaloids: dihydroergocornine (DHCO), dihydroergocristine (DHEC), alpha-dihydroergocryptine (α-DHC), and beta-dihydroergocryptine (β-DHC). The drug was developed in the 1940s by Albert Hofmann [138], and thus, is one of the oldest nootropic drugs still in use. It was initially used against hypertension [139], but later was found, by chance, to improve mental health when patients with Alzheimer’s disease were treated for hypertension [140,141]. 

Dihydroergotoxine increased neuronal metabolism, and, in rats, it stimulated local glucose utilization in those parts of the brain related to learning and memory [142]. A similar effect has been observed in patients aged 74 to 79 years with multi-infarct dementia [143]. Dihydroergotoxine modulated synaptic neurotransmission in the brains of elderly rats by reducing levels of monoamine oxidase enzymes, which are commonly elevated in aging. Monoamine oxidases degrade neurotransmitters and are essential for normal brain metabolism, but an age-related increase in their activity can deplete catecholamine neurotransmitters (dopamine, norepinephrine, and adrenaline), which impairs mental function [144,145]. In a rat experiment, hydergine regulated the release of the neurotransmitter acetylcholine from the hippocampus [146] and increased the number of cholinergic receptors [147]. Furthermore, dihydroergotoxine slowed the release of lipofuscin, which has been associated with the aging process of neurons in old rats [148]. Hydergine acts as a peripheral and cerebral vasodilator. In monkeys, it increased blood flow and oxygen consumption through the brain [149].

Dihydroergotoxine also protects the brain against hypoxia. In a double-blind placebo-controlled quantitative EEG and psychometric study, volunteers inhaled a combination of gases simulating high altitude conditions, which caused hypoxia that led to reduced alertness, intellectual function, and performance depending on reaction time. However, after oral administration of hydergine, subjects who were again exposed to the same conditions achieved significantly better results [150]. It is used mainly in Alzheimer’s disease and vascular and post-traumatic dementias in old age [151,152]. 

It is non-toxic and relatively safe, with possible side effects including nausea, indigestion, orthostatic hypotension, and blurred vision. It is contraindicated in hypotension, psychosis, and a slow heartbeat. In practice, low dosing is common. The recommended daily dose is up to 6 mg [153]. The combination with piracetam and xanthine derivatives, which have a bronchodilator and vasodilatory effect, increases the effect of ergot alkaloids [154,155].

## 5. Cholinergics

Substances belonging to this group usually include acetylcholine precursors or cofactors of its formation. Acetylcholine is the primary mediator in processes related to memory, thinking, counting, and attention. The important representatives of this group include acetyl-L-carnitine, which is a source of acetylcholine precursors, acetyl, choline, lecithin, and pyrrolidine derivatives [156,157]. These substances are classified as cognitive substances that primarily affect cholinergic transmission in the brain rather than nootropics [158], so only their well-known representative, phosphatidylcholine (lecithin), is described in more detail.

### Phosphatidylcholine (Lecithin)

**Figure 9:**
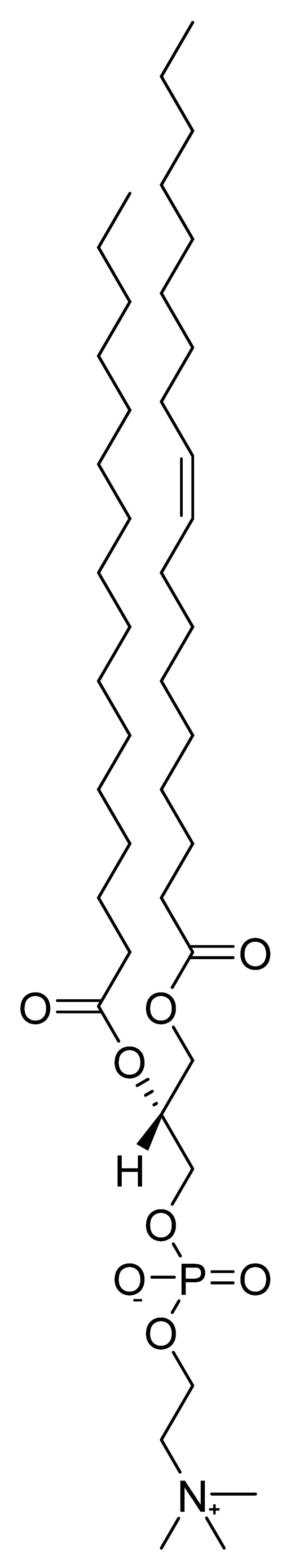
Chemical structure of phosphatidylcholine.

Phosphatidylcholine (Figure 9) belongs to a group of compounds called phospholipids, which are the main lipid components of cell membranes. A mixture of these phospholipids in oil is referred to as commercial lecithin. The abundant component of lecithin-containing supplements is phosphatidylcholine, followed by phosphatidylethanolamine, phosphatidylserine, phosphatidylinositol, and phosphatidylglycerols. Fatty acids ester-linked to phosphatides are represented in lecithin by palmitic, oleic, and linoleic acids [159]. The primary source of commercial lecithin today is soybean and sunflower oil. Lecithin is also present in egg yolk, liver, whole grain products, and nut kernels [160,161]. 

The presumed mechanism of action shows choline being slowly released from lecithin as a precursor for acetylcholine synthesis. Although the mechanism of action of lecithin appears to be clear, the results of tests of its effectiveness in clinical trials were less convincing. Results of an in vivo experiment suggested that the administration of phosphatidylcholine to mice in a model of dementia increased acetylcholine concentrations in the brain and improved memory [162]. 

In contrast, results from randomized in vivo studies have shown no benefit of lecithin in treating patients with Alzheimer’s and Parkinson’s disease [163]. The data suggest that a lack of control over the subject’s learning levels may contribute to inconsistent findings. Phosphatidylcholine supplements may not uniformly improve memory, suggesting that the dose and time parameters required to achieve a therapeutic effect may depend on variables intrinsic to individual subjects. Students that have subnormal endogenous choline levels, may see a greater increase from phosphatidylcholine supplements, resulting in a measurable improvement in explicit memory, compared to healthy subjects with normal endogenous choline levels [164]. 

Many signs of aging are probably related to the fact that the older a person is, the higher the concentration of lecithin in the blood is needed to produce a good effect. The recommended dose of lecithin for prevention is 1200 mg three times a day. In patients, the amount should be 10–15 g/day or higher [160]. 

## 6. Plants and Their Extracts with Nootropic Effects

Pharmaceutical companies invest vast sums of money in discovering substances that could be used in the future to alleviate or treat mental disorders affecting people worldwide. The potential beneficial substances from plants, known as phytochemicals, are still being explored. Several species of plants have been selected for testing as nootropic agents because of their use in traditional medicine, and research has already identified several promising natural substances that could act as cognitive enhancers (Table 1) [3,165,166].

### 6.1. Herbal Drug Plant Collection

Despite the continuous improvement in the production of synthetic drugs, herbal compounds still have considerable use, but the important ones come from commercial plantings. The consumption of popular species is high, and it is impossible to cover it only by collecting wild plants. Bred varieties generally provide higher yields and reduce the risk of confusion or falsification [40,41]. Nowadays, the collector often encounters difficulties that were not present before, such as chemical damage or contamination of the growing plants [167]. The composition and total content of complex active constituents are variable during plant development and vegetative growth. Choosing a suitable period for harvesting or collecting is essential. Plants should not be harvested in humid or rainy weather, but only when dry. During harvesting, the plants must not be damaged because breaking the leaves sometimes affects the active compounds in an undesirable manner. Many compounds, such as vitamin C or tannins, can also react with metals. Therefore, if possible, the plants or parts are collected in their entirety. The leaves and stems are usually harvested just before flowering or during flowering. The flowers are harvested shortly before full development, but some may still be in the bud stage. The fruits and seeds are harvested at the time of full ripeness. For roots and rhizomes, the suitable period is the developmental dormancy of the plant, which is usually autumn or spring. The bark is harvested in the temperate zone at the beginning of vegetative growth in early spring, or, in the tropics, throughout the year [168,169].

### 6.2. Plant Material Processing

The extraction of plant material to produce an effective drug usually involves many technical steps. Contaminants on aboveground parts can be removed by sieving or winnowing, while underground organs are decontaminated by washing and brushing. The common method of preservation continues to be drying, preceded by fermentation in some cases. By removing water, enzymes are inactivated, and the growth of fungi and bacteria is limited [170,171]. Most plants should be dried in the shade, and the temperature should not exceed a specific limit. For plants containing volatile essential oils, this limit is 40 °C [172,173]. Freeze-drying or lyophilization is also frequently used. For this method, fresh plant material is rapidly frozen at a temperature of −20 °C to −50 °C and then dried under a high vacuum. However, in some cases, freeze-drying imperfectly preserves important classes of medicinal compounds such as phenolics and volatile substances, reducing the effectiveness of some plant drugs. Additionally, the material dried in this way is very hygroscopic [174,175]. Freeze-dried drugs have to be stored away from moisture, as well as dust, insects, and light [172,173]. The drug is usually processed or cut after drying. Active compounds are used either directly in the form of medicinal products or indirectly as raw materials to obtain active compounds, which become part of medicinal products. Medicinal products produced directly from drugs can be in the form of tea for water infusion, granules, tablets, extracts, and divided or undivided powders. Active compounds are obtained from herbal materials often by extraction (alkaloids, glycosides), distillation (essential oils), or pressing (oils, fats) [176,177]. 

**Table 1:** Phytochemicals with potential nootropic effect.

Ref.	Phytochemical Group	Main Active Compounds	Uses and Effects	Botanical Name
[178,179,180,181,182]	Terpenoids	Panaxosides (Ginsenosides)	Adaptogen, antioxidant, vasorelaxation	*Panax ginseng*
[183,184,185,186,187]		Ginkgolides	Antioxidant, neuroprotection, vasodilatation	*Ginkgo biloba*
[188,189,190]		Asiatic acid, centellic acid, madecassic acid, asiaticoside, centelloside, madecassoside, brahmoside	Antioxidant, anxiolytic, nootropic	*Centella asiatica*
[191,192,193,194]		Withanolides	Antioxidant, increase in red blood cell content, nootropic	*Withania somnifera*
[195,196,197,198]		Bacosides, bacopasides	Antioxidant, cognitive enhancer, neuroprotectant	*Bacopa monnieri*
[199,200]	Alkaloids	Methylxanthines	Anxiolytic, nootropic, panicolytic, stimulant	*Paullinia cupana*
[201,202,203]	Polyphenols	Rosavins, salidroside	Adaptogen, antidepressant, antioxidant, anxiolytic, stimulant	*Rhodiola rosea*
[204,205,206]		Schisandra lignans	Antioxidant, neuroprotection	*Schisandra chinensis*
[207,208,209,210,211]	Diverse ^1^	Eleutherosides, ciwujianosides	Antioxidant, memory improvement	*Eleutherococcus senticosus*
[212,213,214]		Macamides, macaenes	Antioxidant, antidepressant, cognitive enhancer	*Lepidium meyenii*

^1^ Heterogenous group of chemical compounds.

### 6.3. Specific Plant Species

#### 6.3.1. Ginseng (*Panax ginseng*)

As a drug, ginseng is prepared in two different ways, which affect the content of active components and the degree of medicinal effects. It can be modified either by peeling and drying the root, after which it is called white ginseng, or the root can be steamed without peeling when it is referred to as the “hotter” red ginseng [215]. 

Ginsenosides have been shown to stimulate nitric oxide (NO) production in several systems. Purified ginsenoside Rb_1_ induced NO production in human aortic endothelial cells in vitro. The effect on the NO pathway is responsible for ginseng’s vasorelaxant and mildly hypotensive effect [182].

Ginseng increased the activity of the antioxidant enzymes superoxide dismutase and glutathione peroxidase in rats in vivo. Thus, supplementation may prevent increased oxidant accumulation and age-related oxidative protein and nucleic acid damage [178]. Experimental data from tests on male chicks suggest that Rb_1_ may improve memory for the task of visual discrimination and that the nootropic effect may be associated with changes in anxiety [179]. Ginsenoside Rb_1_ also reduced simulated Alzheimer’s disease in a rat model. Thus, it could be used in the future as a therapeutic agent for patients with memory impairment [180]. Ginsenoside Rg_1_ supplementation improved the performance of old mice in the behavioral test, significantly increasing the expression of proteins associated with synaptic plasticity in the hippocampus, including synaptophysin and *N*-methyl-D-aspartate receptor subunit 1 [181]. Oral administration of a combination of *Ginkgo biloba* and *Panax ginseng* extracts improved memory in rats. Data on test drug effects suggested the involvement of a serotonergic transporter as an important neurochemical correlate of rat behavior and memory effects of study drugs [216]. 

Ginseng’s effect on the human body can be described as adaptogenic. It increased the physical and mental resilience of the organism, eliminated fatigue, and helped the body to adapt to any current needs [217]. It is recommended to use a standardized ginseng extract at a dose of 200 mg per day ginseng for an extended period of time. Standardization refers to the content of ginsenosides, which usually ranges from 1.5 to 7 percent. Alternatively, 0.5 to 2 g of dry root per day is recommended, with ginseng taken in tea or chewed [218]. Ginseng is contraindicated in patients with acute asthma and hypertension. In large doses, it can cause excessive body stimulation, restlessness, insomnia, increased blood pressure, nervousness, inability to concentrate, headaches, and nosebleeds [218,219].

#### 6.3.2. Ginkgo (*Ginkgo biloba*)

The leaves and ripe fruit are harvested from spring to early autumn. Leaves are used to make alcohol extracts (tinctures) or dried and ground [220]. Hulled and roasted ginkgo kernels are also consumed [221]. Mechanisms of action of *Ginkgo biloba* compounds include free radical scavenging for antioxidant activity, antagonistic effects on platelet-activating factor, vasodilation, and an overall reduction in blood viscosity [183,187]. 

Results of an ex vivo rat experiment showed that *Ginkgo biloba* extract had specific neuroprotective effects that may be useful in treating chronic cerebral hypoperfusion. The extract’s pharmacological mechanism involved modulating inflammatory mediators and the cholinergic system [184]. The triterpene lactones (ginkgolides A, B, C, and bilobalide) in the *Ginkgo biloba* extract have antioxidant, anti-inflammatory, and neuroprotective effects. In addition, in an experiment on mice, the extract had an antagonistic effect on glycine and GABA type A receptors [185]. 

A double-blind, placebo-controlled clinical trial in which participants received validated neuropsychological tests before and after treatment with *Ginkgo biloba* extract indicated significant improvement in working memory and information processing speed [186]. In contrast, a critical review of the evidence from several randomized clinical trials did not provide convincing evidence that *Ginkgo biloba* extracts taken either in a single dose or over a long time had a positive effect on any aspect of cognitive performance in healthy human subjects under sixty years of age [222]. 

Still, *Ginkgo biloba* extracts are widely prescribed to treat cerebral dysfunction and neurological disorders. Doses of 120–300 mg of standardized *Ginkgo biloba* 761 extracts (24% flavone glycosides and 6% terpene lactones) per day should be administered [183,223,224]. No side effects have been reported at regular doses, but mild stomach irritation and headaches occasionally occur with excessive consumption. It causes blood thinning, so people taking some anticoagulants should not take the drug before surgery [219,225].

#### 6.3.3. Asiatic Pennywort (*Centella asiatica*)

Centella’s use in traditional medicine is diverse and varies regionally. In the countries of origin, fresh leaves are consumed as a salad, as part of curry spice mixes, or cooked as a vegetable [226]. 

An ethanol extract of *C. asiatica* mediated protection against amyloid-β-induced aggregated neurotoxicity by modulating the antioxidant defense system in cells in vitro, including superoxide dismutase, catalase, glutathione peroxidase, glutathione reductase, and glutathione and glutathione disulfide levels. *C. asiatica* is a traditional medicinal herb with strong antioxidant activity that reduces amyloid-β deposition in the brain. Amyloid-β is the major component of senile plaques and neurofibrillary tangles found in the brains of patients with Alzheimer’s disease. This highlights the potential therapeutic and preventive value of *C. asiatica* in treating Alzheimer’s disease [189]. 

The results from in vivo experiments on rats in a maze, monitoring social interactions, locomotor activity, and cage tests, showed that pure asiaticoside, and methanol or ethyl acetate extracts of *Centella asiatica* had anxiolytic activity. In addition, asiaticoside did not affect locomotor activity, suggesting that this compound does not have sedative effects [227]. Another in vivo study in mice revealed that a NO modulating mechanism may be involved in the protective effect of *Centella asiatica* against anxiety caused by sleep deprivation, oxidative damage, and neuroinflammation [188]. A study in juvenile and young adult mice demonstrated the nootropic effect of an aqueous extract of *C. asiatica*. Treatment resulted in increased hippocampal acetylcholinesterase activity and dendritic arborization of hippocampal CA3 neurons. Thus, treatment with *C. asiatica* during the postnatal developmental stage can affect neuronal morphology and support brain function [190]. 

The reported typical daily dose of *C. asiatica* is approximately 600 mg of dried leaves, or from 60 mg to 120 mg of standardized extract of *C. asiatica* (contains at least 85% of triterpenoid glycosides) [228,229]. 

Based on clinical studies, the reported tolerability of orally administered extracts of *Centella* is high, and no interactions with other drugs are known. Although no teratogenic effects have been reported, the drug should not be used during pregnancy and lactation. Use by children is also not recommended [230].

#### 6.3.4. Ashwagandha (*Withania somnifera*)

The roots and the leaves are harvested and used mainly in dried form. An infusion is prepared from the leaves and a decoction from the root. Fruit is sometimes used as an emetic [231]. 

A study in mice indicated that ashwagandha increased the content of hemoglobin, platelets, and red as well as white blood cells. An increase in red blood cells increases the blood’s ability to transport oxygen to the peripheral system, ensuring greater maximum aerobic capacity [194]. In another study, rats were used as a model of tardive dyskinesia, a disorder characterized by involuntary neurological triggering that leads to spontaneous, repetitive body movements, such as grimacing, sticking out the tongue, or lip movements that were simulated by injection of reserpine. Oxidative stress and lipid peroxidation products are involved in the pathophysiology of this disease. Long-term administration of *Withania somnifera* root extract to the rats significantly reduced lipid peroxidation, restored reduced glutathione levels, and reversed the decrease in brain superoxide dismutase and catalase levels induced by reserpine treatment. Thus, *Withania somnifera* root extract could be a helpful drug for treating drug-induced tardive dyskinesia [192]. Several tests in animal models have confirmed the nootropic effect of ashwagandha and its potential as a treatment for Alzheimer’s disease [191,193]. In another study, the steroidal lactone withaferin, a bioactive compound from the group of withanolides showed significant anticancer properties both in vitro and in vivo [232]. 

The dosage can be from 6 to 10 g of ground roots of ashwagandha per day or the equivalent of 750 mg to 1250 mg of extract per day [233]. Ashwagandha is not recommended in cases of hyperthyroidism or pregnancy. It is a relatively safe drug when used at the recommended doses. Overdoses can cause gastrointestinal problems and vomiting; therefore, treatment should be started with small doses and gradually increased. Ashwagandha is best taken in the evening because, in substantial doses, the herbal extract can act as a sedative [234].

#### 6.3.5. Water Hyssop (*Bacopa monnieri*)

In countries of natural occurrence, it is sometimes used as a leafy vegetable in salads or soups [235]. 

In vitro treatment of rat astrocytes with methanol extract of *Bacopa monnieri* significantly reduced damage caused by high NO concentrations. It has been suggested that glial cells may produce NO by an enzyme-independent mechanism when stimulated by superoxide radicals, and the study results verified the antioxidant activity of Brahmi plant extract [196]. 

Treatment of albino rats with an alcoholic extract of *Bacopa monnieri* increased protein kinase activity and caused an increase in protein in the hippocampus. Overall, the extract has improved learning ability by enhancing cognitive function and memory retention. The chemical compounds responsible for this facilitating effect have been identified as a mixture of two saponins, bacosides A and B [197]. Choline acetyltransferase expression in the hippocampus was studied in olfactory bulbectomy mice compared to controls. Olfactory bulbectomy reduced cholinergic activity and thus choline acetyltransferase expression in the hippocampus. However, subsequent administration of *Bacopa monnieri* alcohol extract reversed this effect and gradually improved the induced cognitive dysfunction [195]. In a rat model of Alzheimer’s disease, *Bacopa monnieri* alcohol extract improved escape latency in the Morris water maze test. In addition, the loss of neurons and the density of cholinergic neurons were also mitigated [198]. Experiments have shown inhibition of the degeneration of cholinergic neurons by *Bacopa monnieri*, suggesting that the herb is a cognitive enhancer and neuroprotectant and may serve as a potential adjunctive drug for treating Alzheimer’s disease [195,198]. 

The *Bacopa monnieri* liquid extract dosage (ratio 1:2) is 5–12 mL per day for adults and 2.5–6 mL per day for children aged 6–12 years. For *Bacopa monnieri* extracts standardized at 20% content of bacosides A and B, 200–400 mg in divided doses for adults and 100–200 mg daily in divided doses for children is recommended [236,237].

No serious side effects have been reported. Rarely, mild sedation or digestive problems may occur after ingestion [238,239].

#### 6.3.6. Guarana (*Paullinia cupana*)

The seeds, the so-called guarana nuts, are harvested at full maturity. They are first roasted, then sifted by sieving, mechanically crushed, and mixed with water to make a bitter paste with high caffeine content. A coffee-like beverage is prepared by simmering guarana paste with hot water. Guarana paste is also added to syrups, and various non-alcoholic and alcoholic drinks are prepared from it, mainly popular in Brazil. Sometimes, the guarana paste is dried, ground into a powder, and used to make tablets [240,241]. 

In an in vivo study, the aqueous fraction of *Paullinia cupana* seeds was repeatedly administered to rats who were then placed in a T-maze, a model of generalized anxiety and panic disorders, and the guarana was shown to have anxiolytic and panicolytic effects [242]. The impact of long-term administration of *Paullinia cupana* seed extract by gavage to rats at various doses on their cognitive behavior was studied using the Morris water maze test, which showed identical results in rats with scopolamine-induced amnesia compared with controls [200]. Mice that ingested guarana suspension showed a significant increase in physical capacity when exposed to stressful situations such as forced swimming. After both single and chronic administration, guarana partially reversed the amnesic effect of scopolamine, as measured by a passive avoidance test in rats and mice, indicating a positive impact on memory acquisition [199]. Studies have shown that oral administration of processed *Paullinia cupana* seeds had a significant nootropic effect. Herbal drugs that exhibit this property may offer a useful adjunct therapeutic option for preventing or treating memory deficits, such as those seen in Alzheimer’s or Parkinson’s disease [199,200]. 

A typical dose is 75 mg of guarana extract (approximately 12% caffeine) administered as a tablet [243]. Guarana should not be used in persons with cardiovascular disease, who are pregnant or breastfeeding, have chronic headaches, diabetes, insomnia, mental disorders, stomach ulcers, or are taking theophylline [244]. 

#### 6.3.7. Eleuthero (*Eleutherococcus senticosus*)

The root is ground to a powder and formed into tablets or used in the form of a tincture. Infusion of the above-ground parts is also sometimes used [245]. 

In vitro experiments showed the antioxidant and antiradical activity of eleuthero [208], including the inhibition of lipid peroxidation [207]. 

In an in vivo study, an aqueous extract of eleuthero reduced acute stress in mice [210]. A study in normal mice examined the effects of an aqueous extract from eleuthero leaves on memory function. These in vivo tests showed that oral administration of the extract improved memory functions, and ex vivo confirmed that the active compounds of the extract, such as eleutheroside M and ciwujianoside B and C3, were able to penetrate the BBB and act on the brain. These three compounds and the leaf extract showed dendritic elongation activity against primary cultured cortical neurons, which may be related to improved memory [211]. 

Tests on healthy volunteers have also concluded that the active compounds of eleuthero affect cell defense, physical fitness, and lipid metabolism [209]. The detoxification properties of the extract have been used in treating chronic lead poisoning in mine workers [246]. Siberian ginseng has also been used in cosmetics [207]. 

The recommended daily dose of eleuthero is 2–3 g of dried root or an equivalent preparation [247]. According to the Russian Pharmacopeia, a standardized liquid extract of roots and rhizomes of *Eleutherococcus senticosus* (10 mg of the extract is equivalent to 120 mg of the crude herb) is currently available as an over-the-counter drug in a ratio of 1:1 with 40% ethanol. In the Russian medical system, this extract is recommended for oral use at a daily dose of 20–40 drops for an adult. However, further research is needed to investigate the appropriate dosing regimen to improve healthy adults’ cognitive function and physical performance [246,248]. Side effects occur infrequently. Eleuthero increases blood pressure, so its use in hypertension is not recommended [249]. 

#### 6.3.8. Rhodiola (*Rhodiola rosea*)

Rhizomes and roots from older plants are collected, dried, and subsequently used for extract preparation [250]. 

According to an in vitro study, salidroside, a phenylpropanoid glycoside isolated from *R. rosea* L., showed a protective effect in cultured PC12 neuronal cells against hypoglycemia and serum-restricted cytotoxicity, probably through modulation of gene expression associated with apoptosis, restoration of mitochondrial membrane potential, and inhibition of intracellular oxygen radical production [203]. 

An in vivo study was performed to investigate the effects of a single oral dose of an aqueous-alcoholic extract (plant material was extracted with 2% ethanol diluted with tap water) of *R. rosea* containing 3% rosavin and 1% salidroside on CNS activity in mice. The extract was tested for adaptogenic, antidepressant, anxiolytic, nociceptive, and locomotor activity at various doses using predictive behavioral tests in the animal model. The results showed that this extract significantly induced adaptogenic, antidepressant, anxiolytic, and stimulating effects [202], but the effects were not dose-dependent. 

In a different trial, the effect of *R. rosea* L. extract on mood, anxiety, stress, and cognition in moderately anxious students was evaluated. Compared with the control, the experimental group showed a significant reduction in anxiety, stress, anger, confusion, and depression, and an improvement in general mood after treatment for two weeks. However, no significant difference in cognitive performance was observed between the groups [201]. 

The optimal dose of rhodiola extract for long-term use was 100–170 mg per day, and the rosavin content of the extract should be 3.6–6.14 mg per weight of the extract. This would suggest a daily dose of roughly 360–600 mg of standardized *Rhodiola rosea* extract containing 1% rosavin [251].

No serious side effects have been identified so far. Because it affects human nature, it is not recommended for patients who have manic–depressive psychosis. Rhodiola should also not be used by children, pregnant and breastfeeding mothers, or people with high blood pressure [252].

#### 6.3.9. Schisandra (*Schisandra chinensis*)

The often used parts are fruits and seeds. A tincture can be prepared from crushed seeds and a tea brewed from dried berries, shoots, and leaves. The fruits are consumed dried or marinated in sugar or honey to make jam, syrup, juice, or compote. They can also be stored frozen. In addition to syrups and juices, a strong sweet wine can be made from the juice of the berries [253,254]. Schisandra fruits are known to the people of the Far East primarily as a tonic and stimulant against fatigue and exhaustion [253]. 

An in vitro study was performed to determine the neuroprotective effects of dibenzocyclooctadiene lignan, schisantherin A, from the fruits of *Schisandra chinensis* against selective dopaminergic neurotoxin 6-hydroxydopamine-induced neural damage in human neuroblastoma cells. Pretreatment with schisantherin A provided neuroprotection against induced cytotoxicity, regulated the intracellular accumulation of reactive oxygen species and inhibited NO overproduction by reducing the overexpression of inducible nitric oxide synthase in cells [206]. 

In other in vitro and in vivo experiments, SH-SY5Y (human neuroblastoma) cells were incubated with 1-methyl-4-phenylpyridinium ion, and mice treated with 1-methyl-4-phenyl-1,2,3,6-tetrahydropyridine were used to determine neuroprotection of schisantherin A. Pretreatment with schisantherin A significantly inhibited the induced cytotoxicity in SH-SY5Y cells. In addition, schisantherin A provided significant protection against induced dopaminergic neuronal loss in a mouse model of Parkinson’s disease [204]. These findings demonstrate that schisantherin A may have potential therapeutic value for oxidative stress-related neurodegenerative disorders, such as Parkinson’s disease [204,206]. 

In vivo cognitive tests such as the Morris water maze and the passive step-down avoidance tests were performed with rats given oral doses of aqueous or 95% ethanolic extract of *Schisandra chinensis* (petroleum ether fraction) and showed that the extract could partially reverse the effects of decreasing activity of superoxide dismutase, catalase and the overall antioxidant effect induced by D-galactose, and to maintain normal levels of glutathione, malondialdehyde and nitric oxide in serum, prefrontal cortex, striatum, and hippocampus. The extract improved the overall induced cognitive deficit [205]. 

The optimal dose of dried schisandra fruit for human administration is 2–6 g per day. For an average human body weight of 60 kg, the dose is 0.03–0.1 g of fruit per kg of body weight [253,255]. No serious side effects have been reported. Side effects have only occurred after regular ingestion of excessive amounts of fruits and included restlessness and insomnia [256].

#### 6.3.10. Maca (*Lepidium meyenii*)

Maca root is consumed either fresh or dried and has a distinctive taste and aroma. In South America, a sweet porridge or pudding called *mazamorra de maca* is made from dried roots, while the fresh root is cooked like potatoes. It can also be ground into flour, with a composition similar to cereal grains. A slightly alcoholic beverage called *maca chica* is made from the maca plant. Many growers mix and grind the leaves with the roots [257,258]. 

Polysaccharide fractions from maca leaves showed different in vitro scavenging capacities on 2,2-diphenyl-1-picrylhydrazyl, hydroxyl, and superoxide anion radicals [212]. 

Researchers have recently been interested in the neuroprotective effects of *Lepidium meyenii*. Experiments in vivo and ex vivo tests have shown the effect of *Lepidium meyenii* in reducing latency in untrained and trained mice. In the swimming strength test, maca shortened the immobility time. It also increased the uterine weight of mice after ovariectomy. *Lepidium meyenii* appeared to positively affect latent learning in ovariectomized mice and exhibited antidepressant activity [214]. Maca improved cognitive function, motor coordination, and endurance in middle-aged mice, increased mitochondrial respiratory function, and upregulated proteins associated with autophagy in the cortex [213]. 

These findings suggested that maca might be an effective functional food to slow age-related cognitive decline. The optimal dose has not been determined; however, the amount of maca root powder used in many studies was in the range of 1.5–3 g per day for the average human adult [259,260].

So far, no serious side effects or contraindications to the extracts have been reported. Maca seems to be safe, effective, and non-toxic [261]. 

## 7. Summary and Recommendations

Nootropics are a heterogeneous group of drugs that affect the metabolism of neuronal cells in the central nervous system. They mainly improve cognitive function, especially in cases where there is damage or degeneration. Most of these substances do not have an immediate effect after a single administration and must be used for some length of time before there is a measurable improvement. They are used in acute, subacute, and chronic conditions of memory, consciousness, and learning disorders and as a supportive treatment in patients with Alzheimer’s disease, schizophrenia, hyperkinetic disorder, or senile dementia. Nootropics are usually very well tolerated. Side effects are rare and typically mild, but some complications can occur. For example, people with cardiovascular disease should not use guarana. This is probably due to the relatively high caffeine content. The available literature suggests that the cardiovascular effects experienced by those consuming up to 600 mg of caffeine per day are, in most cases, mild, transient, and reversible, with no permanent adverse effects [262]. A typical dose of guarana is 75 mg of extract (approximately 12% caffeine) taken as a tablet [243]. Each such tablet, therefore, contains an average of 9 mg of caffeine. Therefore, in order to get close to the limit of 600 mg of caffeine, a person would have to consume around 66 of these tablets per day. A nootropic that could help in this case is naftidrofuryl, which functions as a vasodilator with rheological effects on the blood and is directly used in treating cardiovascular disorders [134]. Some nootropics can also affect psychiatric problems; for example, rhodiola is not recommended for patients with manic-depressive psychosis [252], and dihydroergotoxine is also contraindicated in psychosis [153]. An expert should be consulted before the use of any of these nootropics. Ginseng and eleuthero are contraindicated in patients with hypertension [218,219,249]. Ginkgo causes blood thinning, so people taking certain anticoagulants should not take it, for example, before surgery [219,225]. Additionally, ashwagandha is best taken in the evening because it can act as a sedative in large doses. It is also indicated by its Latin name *Withania somnifera,* where the Latin species name *somnifera* means “sleep-inducing” [234]. Therefore, nootropics users should consider their state of health and mood before deciding to try a certain compound; however, if the recommended dosage is followed, no serious complications should occur. Because of their potential for improving memory and thinking and their easy availability, nootropics have particularly attracted the attention of college students, who call them “smart drugs”. Because of the incomplete clinical evidence on their effectiveness, safety, and social consequences in the case of long-term use, especially with synthetic variants of these drugs, they cannot be recommended to healthy individuals who do not suffer from any cognitive dysfunction. There have not been sufficient experimental studies and results to support prophylactic use, even though the use of herbal supplements with nootropic effects has shown little risk of side effects and contraindications have been minimal. In any case, to be safe, none of these substances should be used during pregnancy or breastfeeding. Future research regarding nootropics should focus on experiments with more diverse human groups, whether in terms of age, health, gender, or weight. It should also mainly focus on young, healthy people, mostly university students, who use these substances a lot and obtain them, especially on the black market. Furthermore, already advanced methods based on neuroimaging assessment should be used more in experiments and studies to confirm or refute the potential beneficial effects.

## Data Availability

Not applicable.
